# 稳定同位素稀释气相色谱-三重四极杆质谱法测定水产品中15种卤代多环芳烃

**DOI:** 10.3724/SP.J.1123.2022.11001

**Published:** 2023-06-08

**Authors:** Xinyu LI, Fang ZHAO, Hua PING, Zhihong MA, Bingru LI, Tingjun MA, Cheng LI

**Affiliations:** 1.北京市农林科学院质量标准与检测技术研究所, 北京 100097; 1. Beijing Institute of Quality Standards and Testing Technology, Beijing Academy of Agriculture and Forestry Sciences, Beijing 100097, China; 2.北京农学院食品科学与工程学院, 北京 102206; 2. College of Food Science and Engineering, Beijing University of Agriculture, Beijing 102206, China

**Keywords:** 同位素稀释法, 气相色谱-三重四极杆质谱, 卤代多环芳烃, 水产品, isotope dilution method, gas chromatography-triple quadrupole mass spectrometry (GC-MS/MS), halogenated polycyclic aromatic hydrocarbons (H-PAHs), aquatic products

## Abstract

建立了测定水产品中15种卤代多环芳烃(H-PAHs)的稳定同位素稀释气相色谱-三重四极杆质谱分析方法,分别对仪器条件和前处理方法进行了优化。在提取样品之前加入同位素内标,校准在前处理过程中待测物的损失,再经加速溶剂萃取提取、凝胶渗透色谱柱和PRiME HLB小柱净化后,采用气相色谱-三重四极杆质谱联用仪测定。采用两根DB-5MS色谱柱(30 m×0.25 mm×0.25 μm),利用微板流路控制技术连接两根色谱柱串联使用,可达到较好的分离效果,且目标化合物峰形良好,响应值高。15种H-PAHs在1~50 μg/L内线性关系良好,相关系数(*r*)≥0.993, H-PAHs相对响应因子(RRF)的相对标准偏差(RSD)值均小于9%,方法检出限为0.009~0.072 μg/kg,方法定量限为0.031~0.240 μg/kg。在空白样品中分别添加0.25、1.0、2.5 μg/kg 3个加标水平的15种H-PAHs混合标准溶液,测定其回收率和精密度,回收率分别为74.6%~116.8%、77.8%~123.2%和71.9%~124.8%, RSD分别为0.6%~8.2%、0.6%~9.0%和0.4%~10.6%。采用所建立的方法对水产品进行检测,实际样品中H-PAHs的总含量为0.60~3.54 μg/kg,其中9-氯菲(9-ClPhe)检出率为100%,且含量最高(1.15 μg/kg)。该方法简化了前处理步骤,具有简便快速、回收率高、稳定性好等优点,适用于实际水产品中H-PAHs的定性、定量分析,为研究H-PAHs在水产品中的残留状况和风险评价提供了可靠的技术支持。

## Abstract (en)

Halogenated polycyclic aromatic hydrocarbons (H-PAHs), including chlorinated polycyclic aromatic hydrocarbons (Cl-PAHs) and brominated polycyclic aromatic hydrocarbons (Br-PAHs), are compounds in which one or more hydrogen atoms replaced by chlorine or bromine atoms. These compounds are not only difficult to degrade but also highly fat soluble and toxic. They are a new type of high-risk organic pollutants with structures similar to those of dioxins, and their toxicity is even higher than that of the parent polycyclic aromatic hydrocarbons (PAHs). The bioaccumulation of H-PAHs can be predicted by their octanol-water partition coefficient (*K*_ow_); in general, higher bioaccumulation capacity and *K*_ow_ values indicate greater fat solubility. Therefore, animal-derived foods with higher fat contents, such as animal meat, milk, aquatic products, and their processed forms, are more likely to be contaminated with higher contents of H-PAHs than those with lower fat contents. In this work, a gas chromatography-triple quadrupole mass spectrometry (GC-MS/MS) method coupled with stable isotope dilution was established to determine 15 H-PAHs in aquatic products. The instrument and pretreatment methods were systematically optimized. The GC-MS/MS used in this method can effectively eliminate matrix interferences and features high sensitivity and low analytical cost; thus, it has good application prospects. The samples were added with an isotope internal standard before extraction to calibrate the loss of the tested substance during the pretreatment process, extracted by accelerated solvent extraction, purified using gel permeation chromatography and PRiME HLB columns, and then analyzed by GC-MS/MS. The use of two DB-5MS chromatographic columns (30 m×0.25 mm×0.25 μm) and microplate fluidics technology to connect chromatographic columns 1 and 2 in series led to better separation effects, good peak shapes, and high target compound responses. The 15 H-PAHs demonstrated good linearities in the range of 1-50 μg/L, with correlation coefficients (*r*) greater than or equal to 0.993. The relative standard deviation (RSD) values of the relative response factor (RRF) of the H-PAHs were less than 9%, the method detection limit (MDL) was 0.009-0.072 μg/kg, and the method quantification limit (MQL) was 0.031-0.240 μg/kg. Three spiked levels of 0.25, 1.0, 2.5 μg/kg were added to the blank samples to determine the recovery and precision. The recoveries for these spiked levels were 74.6%-116.8%, 77.8%-123.2%, and 71.9%-124.8%, respectively, and the corresponding RSDs were 0.6%-8.2%, 0.6%-9.0%, and 0.4%-10.6%, respectively. The total actual content of H-PAHs in aquatic product samples was 0.60-3.54 μg/kg. Among the H-PAHs investigated, 9-chlorophenanthrene (9-ClPhe) showed the greatest detection rate (100%) and highest content (1.15 μg/kg), indicating that H-PAHs widely exist in aquatic products. Thus, further assessment of the dietary exposure risk of these compounds is necessary. The developed method simplifies the pretreatment step, and has the advantages of simplicity, rapid analysis, high recoveries, and good stability. It is suitable for the qualitative and quantitative analysis of H-PAHs in actual aquatic product samples and provides reliable technical support for the residue status and risk assessment of H-PAHs in aquatic products.

卤代多环芳烃(H-PAHs)包括氯代多环芳烃(Cl-PAHs)和溴代多环芳烃(Br-PAHs),是多环芳烃(PAHs)分子中的一个或一个以上氢原子被氯原子或溴原子取代的化合物^[1,2]^,具有难降解、高脂溶性、高毒性等特性^[3]^,是一类与二噁英结构相似的新型高风险有机污染物。H-PAHs的毒性甚至高于母体PAHs,具有致畸性、致癌性和DNA损伤效应,可引起其他不良反应^[4,5]^。

H-PAHs广泛存在于受污染的大气、水和土壤中^[6⇓⇓⇓⇓-11]^,污染来源包括汽车尾气排放、有机物焚烧、工业活动和光化学反应等^[12]^。H-PAHs能够通过多种途径在全球范围内迁移,再经过食物链进行传输富集。人类可通过皮肤接触、呼吸吸入和饮食摄入H-PAHs,其中有80%是通过膳食暴露接触到的,特别是动物源性食物。目前已有文献报道在食品中检测出H-PAHs^[12⇓⇓⇓⇓-17]^。Ding等^[14]^在深圳市场的蔬菜、猪肉和大米中检测出9种H-PAHs的平均含量分别为0.56、4.97和2.75 μg/kg; Tan等^[15]^在鱼样本中检测到3种Cl-PAHs,即9-氯菲(9-ClPhe)、1-氯芘(1-ClPyr)和5-氯苊(5-ClAce),检出含量为0.0059~0.206 μg/kg; Anura等^[12]^在斯里兰卡和日本当地市场的海鲜中检测到Cl-PAHs的含量分别为2.58~27.1 μg/kg和0.35~18.3 μg/kg(干重), Br-PAHs的含量分别为0.30~9.53 μg/kg和0.03~3.34 μg/kg(干重)。

通过辛醇-水分配系数(*K*_ow_)可以预测H-PAHs的生物蓄积,其同类物的*K*_ow_为3.91~6.92,相对应母体PAHs的*K*_ow_为4.02~4.93;而当*K*_ow_为5~7时,生物蓄积能力最强,且*K*_ow_越高表示化合物的脂溶性越强^[13,18]^,所以脂肪含量较高的动物源性食品,如畜肉类、乳类、水产品及其制品等,可能更容易受到H-PAHs的污染。

目前H-PAHs的检测方法有气相色谱-低分辨质谱法(GC-LRMS)、气相色谱-高分辨质谱法(GC-HRMS)和气相色谱-三重四极杆质谱法(GC-MS/MS)。其中GC-LRMS由于动物产品基质复杂,无法得到较好的定性和定量结果;GC-HRMS具有高分辨率和高灵敏度,但价格昂贵,使用并不普及^[19,20]^; GC-MS/MS可以有效排除基质干扰,灵敏度高,分析成本低,具有较好的应用前景^[3,11]^。

本研究采用GC-MS/MS结合稳定同位素稀释技术,建立了一种快速测定水产品中15种H-PAHs的方法,该方法能够校准在前处理过程中待测物的损失,定量准确,可为食品中H-PAHs的风险评估提供技术支持。

## 1 实验部分

### 1.1 仪器、试剂与材料

气相色谱-三重四极杆质谱联用仪(7890B-7000C,安捷伦公司,美国);加速溶剂萃取仪(ASE300,赛默飞世尔公司,美国);氮吹仪(N-EVAP, Organomation公司,美国);真空冷冻干燥机(SCIENTZ-18N,新芝公司,中国);正己烷、二氯甲烷、丙酮(色谱纯,Fisher公司,美国); Oasis PRiME HLB(Waters公司,美国);平行蒸发仪(P-6,步琦公司,瑞士);凝胶渗透色谱(GPC)填料(200~400目,Bio-Beads, Cat #152-2750,Bio-Rad公司,美国), GPC柱(40 cm×2 cm)。

H-PAHs标准品:2-氯蒽(2-ClAnt)、2-溴芴(2-BrFle)、3-溴菲(3-BrPhe)、9-溴菲(9-BrPhe)、9-溴蒽(9-BrAnt)、9-氯菲(9-ClPhe)、1-氯芘(1-ClPyr)、7-氯苯并[*a*]蒽(7-ClBaA)、7,12-二氯苯并蒽(7,12-Cl_2_BaA)、7-溴苯并[*a*]蒽(7-BrBaA)、9,10-二氯蒽(9,10-Cl_2_Ant)、1,8-二溴蒽(1,8-Br_2_Ant)、3-溴荧蒽(3-BrFlu)、9,10-二氯菲(9,10-Cl_2_Phe)、3-氯荧蒽(3-ClFlu)均购自美国Sigma Aldrich公司。7种同位素内标:^13^C_6_-1-ClPyr、^13^C_6_-7-ClBaA、^13^C_6_-7-BrBaA、^13^C_6_-7,12-Cl_2_BaA(美国Cambridge Isotope Laboratories公司)、^13^C_6_-9-ClPhe、^13^C_6_-2-ClAnt、D_9_-9-BrPhe(加拿大Toronto Research Chemicals公司)。图1为15种H-PAHs的化学结构式。

**图1:**
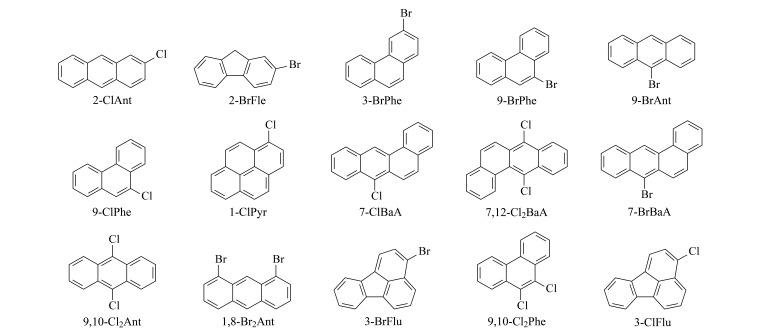
15种H-PAHs的化学结构式

### 1.2 标准溶液的配制

15种H-PAHs、6种净化内标(^13^C_6_-1-ClPyr、^13^C_6_-7-ClBaA、^13^C_6_-7-BrBaA、^13^C_6_-9-ClPhe、^13^C_6_-2-ClAnt、D_9_-9-BrPhe)分别用正己烷配制成1.0 mg/L混合标准储备液,1种进样内标(^13^C_6_-7,12-Cl_2_BaA)用正己烷配制成质量浓度为1.0 mg/L的标准储备液。将15种H-PAHs用正己烷配制成质量浓度为1、5、10、20、50 μg/L的混合标准溶液,加入净化内标和进样内标各5 μL进行测定,7种内标的最终质量浓度为50 μg/L,绘制标准曲线。

### 1.3 样品制备

取水产品样品的可食用部分进行匀浆,冷冻后用真空冷冻干燥机冻干48 h,研磨存放于-20 ℃保存备用。

### 1.4 样品前处理

称取2.00 g冻干研磨后的水产品样品(精确至0.01 g),加入1.5 g硅藻土混匀。在萃取池(34 mL)底部铺1 cm厚硅藻土,将混匀样品填入萃取池,添加5 μL净化内标后,用硅藻土将萃取池填满。用正己烷-丙酮(1∶1, v/v)提取,萃取条件设定:温度100 ℃,加热时间5 min,静态萃取时间5 min,冲洗溶剂体积为池容积的70%,循环次数为3,N_2_吹扫60 s。

将提取液于45 ℃下旋转蒸发至1 mL后,转移至GPC柱中,用150 mL正己烷-二氯甲烷(1∶1, v/v)淋洗,弃去前70 mL淋洗液,收集剩余淋洗液,并于45 ℃旋蒸至近干后用乙腈复溶,上样于PRiME HLB小柱,用10 mL乙腈洗脱,45 ℃氮吹至干后用正己烷复溶,加5 μL进样内标,定容至100 μL后上机测试。

### 1.5 仪器条件

#### 1.5.1 气相色谱条件

色谱分析柱(1和2): Agilent DB-5MS(30 m×0.25 mm×0.25 μm),微板流路控制技术用于连接两根分析柱(1和2)及辅助电子压力控制模块以实现反吹;载气N_2_,纯度≥99.999%,流速1.0 mL/min;进样口温度280 ℃;不分流进样模式;进样量1 μL。升温程序:初始温度100 ℃,保持1 min;以30 ℃/min升温至210 ℃,保持1 min;以2 ℃/min升温至250 ℃,保持1 min;以5 ℃/min升温至260 ℃,保持1 min;以10 ℃/min升温至290 ℃,保持4 min;以3 ℃/min升温至310 ℃,保持3 min;总运行时间为44.4 min。

#### 1.5.2 质谱条件

离子源:电子轰击(EI)源;离子源温度:280 ℃;传输线温度:300 ℃;离子化电压:70 eV;多反应监测(MRM)模式。其余质谱参数见表1。

**表1:** 15种H-PAHs和7种同位素内标的质谱参数

No.	Compound	Retention time/min	Quantitative ion pair(m/z)	Qualitative ion pair(m/z)	CE/ eV	No.	Compound	Retention time/min	Quantitative ion pair(m/z)	Qualitative ion pair(m/z)	CE/ eV
1	2-BrFle	15.698	243.8>165.0	165.0>115.0	30, 40	12	1,8-Br_2_Ant	29.839	336.0>176.0	334.0>176.0	45, 45
2	9-ClPhe	17.595	212.0>176.0	214.0>176.0	40, 40	13	7-ClBaA	35.417	262.0>226.0	264.0>226.0	40, 40
3	2-ClAnt	17.906	212.0>176.0	214.0>176.0	40, 40	14	7-BrBaA	38.419	305.9>226.0	307.9>226.0	35, 35
4	3-BrPhe	20.166	256.0>176.1	256.0>177.1	40, 40	15	7,12-Cl_2_BaA	39.873	296.0>226.0	298.0>226.0	35, 35
5	9-BrPhe	20.454	256.0>176.1	256.0>177.1	40, 40	16	^13^C_6_-9-ClPhe	17.587	218.0>182.1	220.0>182.1	45, 45
6	9-BrAnt	20.982	256.0>176.1	256.0>177.1	40, 40	17	^13^C_6_-2-ClAnt	17.897	218.0>182.1	220.0>182.1	45, 45
7	9,10-Cl_2_Ant	23.114	245.9>176.1	247.9>176.1	35, 35	18	D_9_-9-BrPhe	20.300	264.9>184.1	264.9>186.1	40, 40
8	9,10-Cl_2_Phe	23.513	245.9>176.1	247.9>176.1	35, 35	19	^13^C_6_-1-ClPyr	27.389	242.1>206.0	242.1>207.0	50, 50
9	3-ClFlu	25.384	236.1>200.1	236.1>201.1	45, 45	20	^13^C_6_-7-ClBaA	35.409	267.9>232.0	269.9>232.0	35, 35
10	1-ClPyr	27.392	236.1>200.1	236.1>201.1	45, 45	21	^13^C_6_-7-BrBaA	38.411	311.9>232.0	313.9>232.0	40, 40
11	3-BrFlu	29.058	280.0>201.0	282.0>201.0	30, 30	22	^13^C_6_-7,12-Cl_2_BaA	39.865	301.9>232.0	303.9>232.0	40, 40

CE: collision energy.

## 2 结果与讨论

### 2.1 仪器条件优化

#### 2.1.1 质谱条件优化

通过Scan全扫描筛选出响应高的母离子,再通过子离子扫描得到产物离子,确定出峰时间,优化碰撞能量,选择信号强度最高的两对离子对分别作为定量离子对和定性离子对。质谱参数见表1。

#### 2.1.2 色谱柱优化

已报道文献中的色谱柱常采用30 m色谱柱分离目标物,但由于目标物性质相似,会造成分离效果较差(图2a)。本研究采用两根DB-5MS色谱柱(30 m×0.25 mm×0.25 μm),并利用微板流路控制技术连接两根色谱柱,达到了较好的分离效果(图2b)。

**图2:**
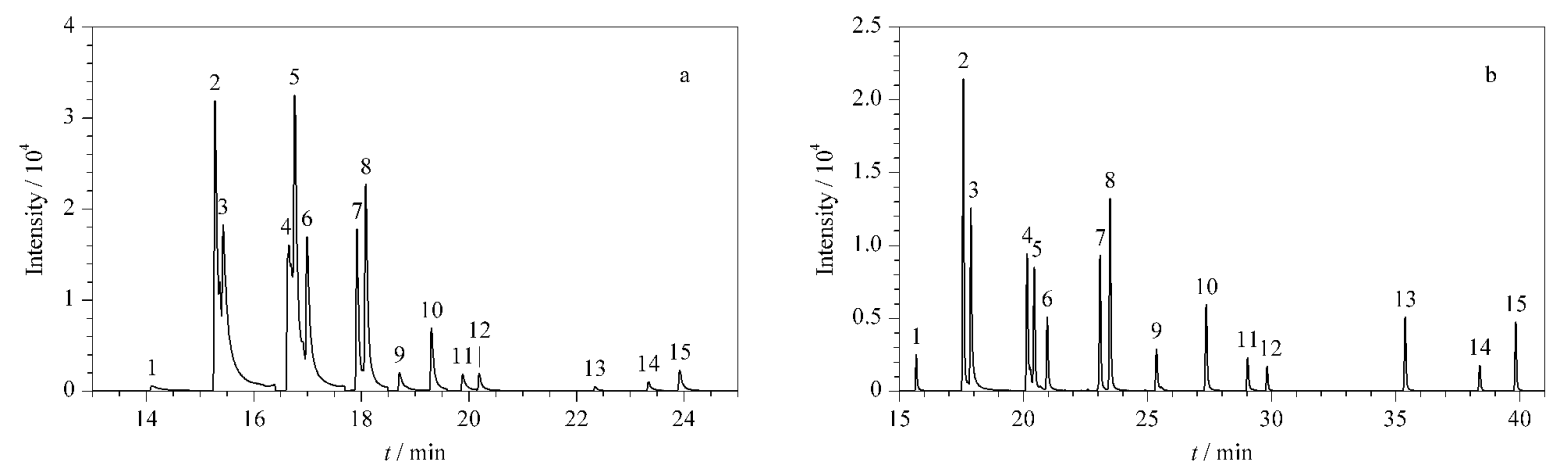
15种H-PAHs混合标准溶液(20 ng/mL)的总离子流色谱图

### 2.2 前处理条件的优化

#### 2.2.1 提取条件的优化

传统提取方法有索氏提取^[21]^、超声提取^[22]^等,但传统方法的溶剂消耗量大且费时费力,因此本方法采用加速溶剂萃取(ASE),耗时短,溶剂消耗量少。本研究分别比较了正己烷、正己烷-二氯甲烷(1∶1, v/v)和正己烷-丙酮(1∶1, v/v)3种提取溶剂对15种H-PAHs的提取效果,3种提取溶剂对15种H-PAHs的提取回收率见图3。其中,使用正己烷-二氯甲烷(1∶1, v/v)和正己烷-丙酮(1∶1, v/v)的回收率普遍高于正己烷,而正己烷-丙酮(1∶1, v/v)的提取效果优于另外两种溶剂,最终选用正己烷-丙酮(1∶1, v/v)作为提取溶剂。之后比较了提取温度(100 ℃和120 ℃)对回收率的影响,100 ℃下的回收率为82%~109%,高于120 ℃下的回收率(54%~91%)。因此最终确定提取条件为:用正己烷-丙酮(1∶1, v/v)于100 ℃下提取,静态提取时间5 min,冲洗溶剂体积为池容积的70%,加热时间5 min,循环次数为3, N_2_吹扫60 s。

**图3:**
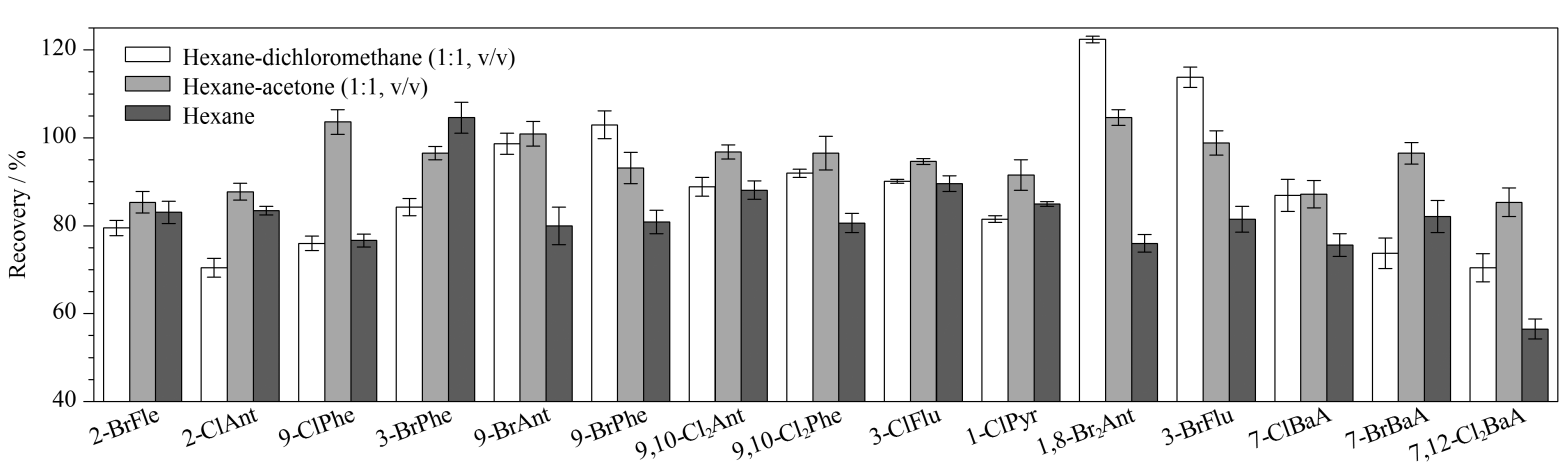
3种提取溶剂对15种H-PAHs回收率的影响(*n*=3)

#### 2.2.2 净化方法的优化

酸性硅胶柱和GPC柱均是常用的净化方法。水产品中含有大量的脂质,使用酸性硅胶会降解含有蒽(Ant)、芘(Pyr)和苯并[*a*]蒽(BaA)等结构的H-PAHs同类物^[3]^,因此本方法采用GPC柱净化。GPC柱用50 mL正己烷-二氯甲烷(1∶1, v/v)预淋洗后,加入样品,再用150 mL正己烷-二氯甲烷(1∶1, v/v)进行淋洗,弃去前70 mL淋洗液,收集后80 mL淋洗液,接收完毕后再用50 mL正己烷-二氯甲烷(1∶1, v/v)冲洗GPC柱。15种H-PAHs在GPC柱上的流出曲线见图4。

**图4:**
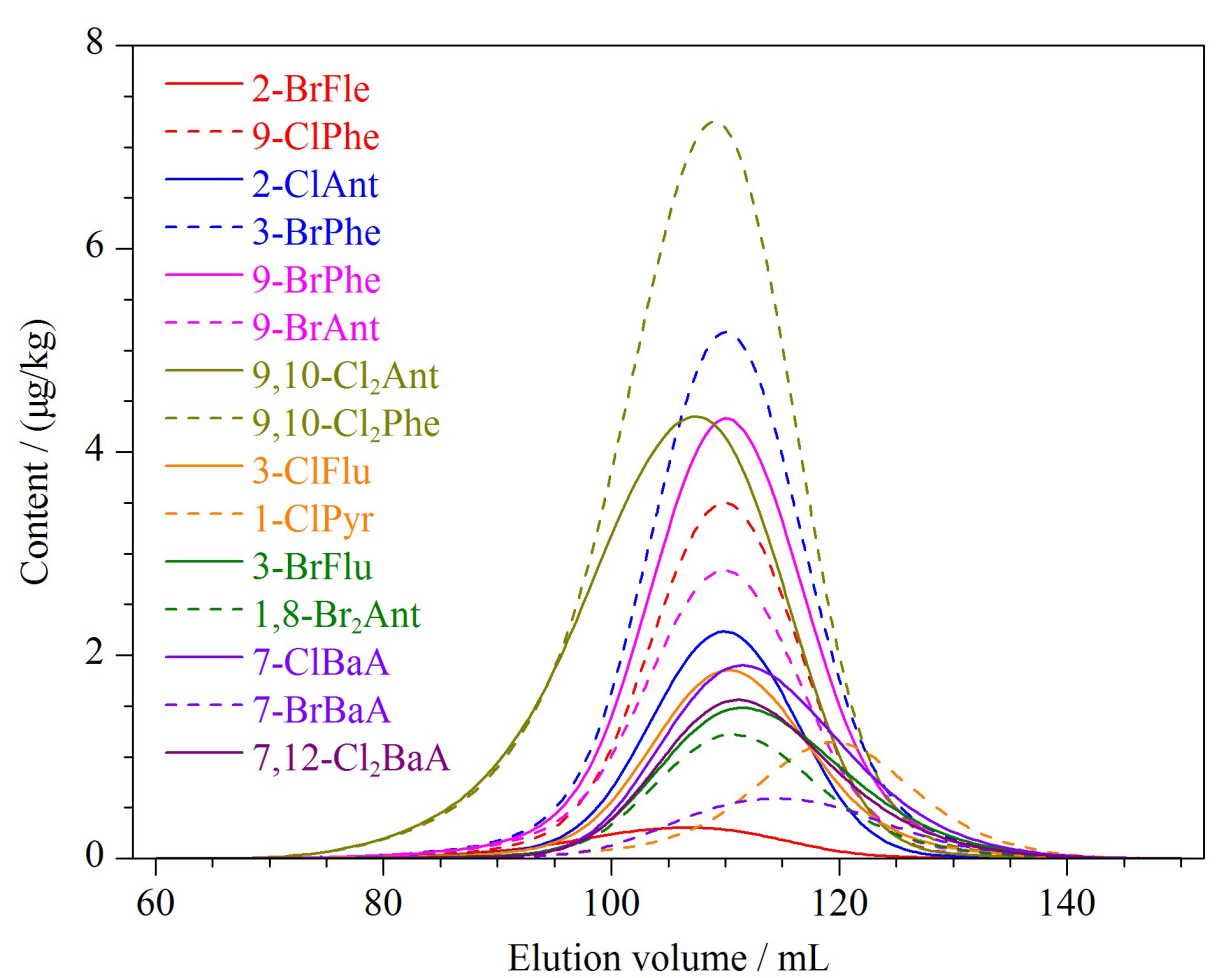
15种H-PAHs的GPC流出曲线

GPC柱可去除大部分脂质,但仍有少量磷脂等脂质干扰物随目标物进入接收液中,于是选用4种固相萃取柱C_18_柱、弗罗里硅土柱(FI)、HLB柱和PRiME HLB柱再次净化,其中PRiME HLB柱去除脂肪和磷脂的效果好。本实验采用乙腈洗脱,4种固相萃取柱对15种H-PAHs的回收率影响见图5。结果表明,HLB柱对H-PAHs的回收率较低,为5%~97%;FI柱和C_18_柱的回收率可分别达到55%~120%和62%~119%; PRiME HLB柱的回收率普遍高于FI柱和C_18_柱,可达88%~106%,且去除脂质效果好,因此选用PRiME HLB柱净化、10 mL乙腈洗脱。

**图5:**
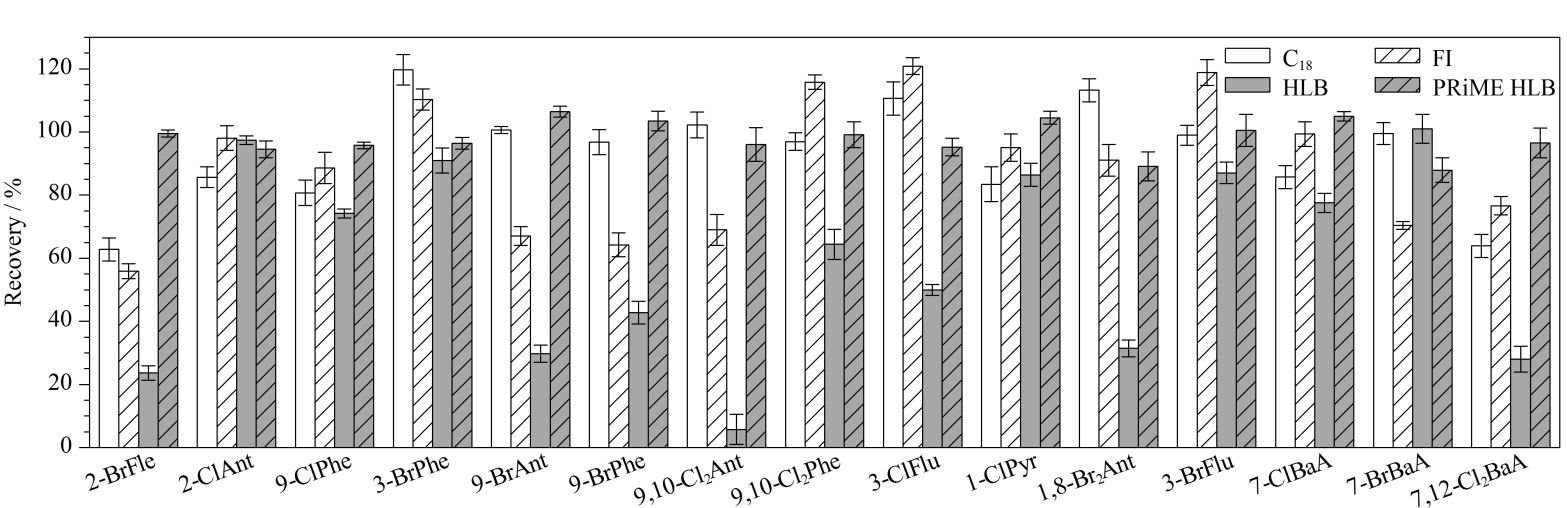
4种固相萃取柱对15种H-PAHs回收率的影响(*n*=3)

### 2.3 基质效应考察

用鲳鱼空白样品的提取液配制基质标准溶液,用正己烷配制溶剂标准溶液,上机测定,得到基质匹配标准曲线和溶剂标准曲线。基质效应(ME)=(基质匹配标准曲线的斜率/溶剂标准曲线的斜率-1)×100%,当|ME|<20%时,表示弱基质效应;当20%≤|ME|≤50%时,表示中等基质效应;当|ME|>50%时,表示强基质效应。结果显示,15种H-PAHs的|ME|为9%~42%,存在一定的抑制或增强效应。因此,本方法通过加入与目标化合物对应的同位素内标以校正基质效应。

### 2.4 方法学考察

#### 2.4.1 线性范围、检出限与定量限

取1.2节中的系列混合标准溶液按上述实验条件和方法进行测定,以目标物与内标峰面积的比值为纵坐标(*y*),目标物与内标质量浓度的比值为横坐标(*x*)绘制标准曲线。15种H-PAHs在1~50 μg/L的范围内表现出良好的线性关系,相关系数(*r*)≥0.993, H-PAHs相对响应因子(RRF)的相对标准偏差(RSD)值均小于9%(见表2)。以3倍信噪比计算方法检出限(MDL),10倍信噪比计算方法定量限(MQL),15种H-PAHs的IDL为0.009~0.072 μg/kg, IQL为0.031~0.240 μg/kg。该结果低于其他使用GC-MS/MS检测得到的检出限^[15,23]^,略高于使用GC-HRMS得到的检出限^[24]^,但GC-MS/MS的分析成本相对较低,具有较好的应用前景。

**表2:** 15种H-PAHs的线性范围、相关系数、相对标准偏差、方法检出限和方法定量限

No.	Compound	Linear range/ (ng/mL)	r	RSD/ %	MDL/ (μg/kg)	MQL/ (μg/kg)
1	2-BrFle	1-50	0.999	3.9	0.012	0.041
2	9-ClPhe	1-50	0.994	5.6	0.055	0.157
3	2-ClAnt	1-50	0.993	8.7	0.009	0.031
4	3-BrPhe	1-50	0.998	5.0	0.015	0.076
5	9-BrPhe	1-50	0.994	7.4	0.027	0.091
6	9-BrAnt	1-50	0.998	5.4	0.040	0.132
7	9,10-Cl_2_Ant	1-50	0.996	4.4	0.015	0.071
8	9,10-Cl_2_Phe	1-50	0.997	4.2	0.019	0.054
9	3-ClFlu	1-50	0.997	3.6	0.072	0.240
10	1-ClPyr	1-50	0.999	3.0	0.014	0.075
11	3-BrFlu	1-50	0.999	7.4	0.018	0.092
12	1,8-Br_2_Ant	1-50	0.995	5.1	0.019	0.058
13	7-ClBaA	1-50	0.997	8.1	0.032	0.092
14	7-BrBaA	1-50	0.996	4.4	0.053	0.167
15	7,12-Cl_2_BaA	1-50	0.998	3.2	0.023	0.112

#### 2.4.2 回收率和精密度

在鲳鱼空白样品中分别添加3个水平(0.25、1.0、2.5 μg/kg)的15种H-PAHs混合标准溶液进行加标回收试验,每个添加水平做6次平行试验,计算平均加标回收率和相对标准偏差,结果见表3。结果表明,15种H-PAHs的平均加标回收率为71.9%~124.8%, RSD为0.4%~10.6%。连续测定3批空白加标样品,每个样品进样6次,15种H-PAHs的批间精密度为0.6%~4.2%,表明本方法的精密度和准确度良好,满足检测条件。

**表3:** 15种H-PAHs的加标回收率和精密度(*n*=6)

No.	Compound	0.25 μg/kg				1.0 μg/kg				2.5 μg/kg		
		Recovery/%	RSD/%	Batch RSD/%		Recovery/%	RSD/%	Batch RSD/%		Recovery/%	RSD/%	Batch RSD/%
1	2-BrFle	75.5	1.6	0.9		110.0	3.8	2.4		99.3	2.4	3.5
2	9-ClPhe	116.8	8.2	1.2		104.7	6.4	3.5		103.1	1.2	4.2
3	2-ClAnt	74.6	2.3	0.7		92.9	5.4	1.2		93.4	10.6	1.7
4	3-BrPhe	85.1	4.0	2.1		121.2	7.3	1.6		124.8	5.2	2.6
5	9-BrPhe	106.5	3.4	0.6		107.8	5.0	1.2		109.6	3.1	3.8
6	9-BrAnt	76.7	2.6	1.3		77.8	2.8	2.4		72.8	8.5	2.1
7	9,10-Cl_2_Ant	101.1	4.6	0.7		101.7	1.9	2.7		91.1	4.0	1.5
8	9,10-Cl_2_Phe	76.9	1.0	1.4		88.8	5.1	1.9		71.9	2.0	1.9
9	3-ClFlu	103.2	0.8	1.3		123.2	1.7	0.8		113.3	1.7	0.8
10	1-ClPyr	96.7	1.0	1.7		94.8	2.4	1.6		91.9	0.4	0.6
11	3-BrFlu	90.1	2.0	2.4		108.2	1.2	2.8		106.3	2.7	2.7
12	1,8-Br_2_Ant	111.9	0.6	1.6		116.8	4.0	3.4		99.6	4.1	3.1
13	7-ClBaA	87.4	3.6	1.8		86.6	3.2	2.4		80.0	2.0	2.1
14	7-BrBaA	82.3	5.2	2.6		88.7	0.6	2.1		82.8	3.2	1.9
15	7,12-Cl_2_BaA	75.6	7.2	3.1		78.1	9.0	0.9		73.9	3.1	1.5

### 2.5 实际样品测定

采集了北京市各农贸市场的10种水产品样品,对其进行检测,各样品中H-PAHs的含量见表4,鲫鱼样品的总离子流色谱图见图6。所有样品均有检出,15种H-PAHs的总含量为0.60~3.54 μg/kg,其中9-ClPhe的检出率为100%。实验结果表明,H-PAHs广泛存在于水产品中,需进一步对膳食暴露风险进行评估。

**表4:** 实际样品检测结果

No.	Compound	Yellow croaker	Crayfish	Turbot	Crucian	Butterfish	Grass carp	Weever	Crab	Carp		Hairy crab	
1	2-BrFle	ND	ND	ND	ND	ND	ND	ND	ND	0.28		ND	
2	9-ClPhe	0.82	0.80	0.69	0.75	0.66	0.78	0.77	1.15	0.53		0.60	
3	2-ClAnt	ND	ND	ND	ND	ND	ND	ND	ND	ND		ND	
4	3-BrPhe	0.26	ND	ND	ND	ND	ND	ND	ND	ND		ND	
5	9-BrPhe	ND	ND	ND	ND	ND	ND	ND	ND	ND		ND	
6	9-BrAnt	ND	ND	ND	ND	ND	ND	ND	ND	ND		ND	
7	9,10-Cl_2_Ant	ND	ND	ND	ND	ND	ND	ND	ND	ND		ND	
8	9,10-Cl_2_Phe	0.38	ND	ND	0.33	ND	ND	ND	ND	ND		ND	
9	3-ClFlu	0.55	ND	ND	0.40	ND	ND	ND	ND		ND		ND
10	1-ClPyr	0.43	ND	ND	0.30	ND	ND	ND	ND		ND		ND
11	3-BrFlu	0.41	ND	ND	0.34	ND	ND	ND	ND		ND		ND
12	1,8-Br_2_Ant	ND	ND	ND	ND	ND	ND	ND	ND		0.36		ND
13	7-ClBaA	0.25	ND	ND	ND	ND	ND	ND	ND		ND		ND
14	7-BrBaA	ND	ND	ND	ND	ND	ND	ND	ND		0.29		ND
15	7,12-Cl_2_BaA	0.44	ND	ND	0.29	ND	ND	ND	ND		ND		ND

ND: not detected.

**图6:**
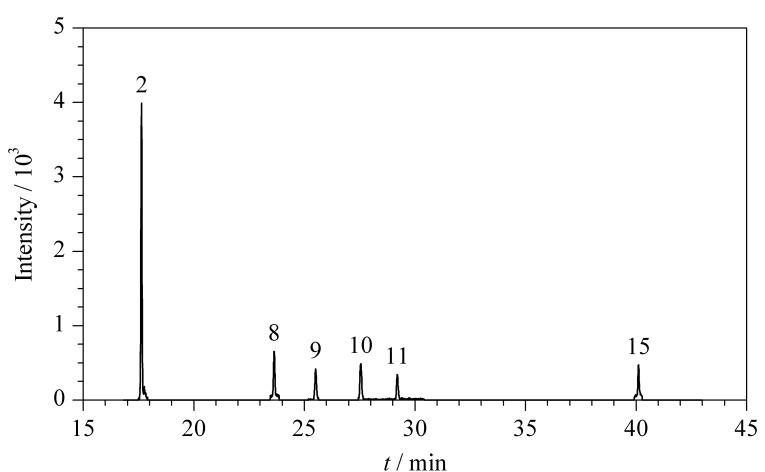
鲫鱼样品的总离子流色谱图

## 3 结论

本研究通过优化加速溶剂萃取、凝胶渗透色谱、固相萃取小柱净化及气相色谱-三重四极杆质谱条件建立了水产品中15种H-PAHs的分析方法。该方法具有灵敏度高、分离效果好等优点,为研究H-PAHs在水产品中的残留状况和风险评价提供了可靠的技术支持。
